# Painful Understanding of VEGF

**DOI:** 10.3389/fphar.2018.01267

**Published:** 2018-11-06

**Authors:** María Llorián-Salvador, Sara González-Rodríguez

**Affiliations:** ^1^Centre for Experimental Medicine, School of Medicine, Dentistry and Biomedical Sciences, Queen's University Belfast, Belfast, United Kingdom; ^2^Instituto de Biología Molecular y Celular, Universidad Miguel Hernández, Elche, Spain

**Keywords:** VEGF, pain, cancer, inflammation, neuropathy

Our nervous system is capable of detecting a wide range of stimuli which can evoke pain. These can generate a short-term sensations (acute pain) which usually resolves. However, sometimes this pain becomes persistent. Constant stimulation provokes alterations in nociceptive transmission, enhancing pain signals and increasing sensitivity. If this state persists for more than 3 months, it is defined as chronic pain. It affects over one-quarter of people worldwide and is more prevalent in women than in men.^1^ The mechanisms that sustain and drive chronic pain have been comprehensively reviewed elsewhere (Woolf and Salter, 2000; Basbaum et al., 2009; von Hehn et al., 2012). The current analgesic treatments (opioids or NSAIDs) do not meet patients needs or are inefficient. In addition, their side effects limit their use. Therefore, the development of new drugs is urgently required. Preclinical research studies have identified an array of molecular targets that are involved in the establishment and maintenance of chronic pain (see references above) and may represent interesting targets for pharmacological intervention. Among these mediators, Vascular Endotelial Growth Factor (VEGF) has been postulated as a key factor.

Alterations in the VEGF system, characterized by changes in the expression of its components, have been related to a plethora of diseases. Some of these diseases can occur concomitantly with pain, such as cancer, rheumathoid arthritis or diabetic complications (Ferrara, 2004; Maharaj and D'Amore, 2007; Rosenstein et al., 2010).

VEGF is a potent pro-angiogenic factor and a key mediator of neovascularisation, a process which is involved in the pathogenesis of cancerous tumors. For this reason, there are currently several anti-VEGF-related drugs used in clinical settings for the cancer treatment in combination with chemotherapy. Anti-VEGF drugs are also used to attenuate neovascularisation in age-related macular degeneration and diabetic macular oedema (Ferrara, 2004; Kim and D'Amore, 2012). However, in recent years, the role of the VEGF family in neuroprotection and nociception has received increased attention (Beazley-Long et al., 2013, 2018; Hulse et al., 2014, 2015, 2016; Selvaraj et al., 2015; Hulse, 2017; Lai et al., 2017). The involvement of VEGF in the pathophysiology of pain is not fully understood, however, the association between this growth factor and some of the main hallmarks of painful diseases warrants the investigation of VEGF as a therapeutic target for pain treatment.

Considering the aforementioned, the potential of VEGF as druggable target for the treatment of different types of pain will be discussed.

The VEGF family consists of five members. The most widely studied of them are VEGF-A and, to a lesser extent, VEGF-B. VEGF-A and VEGF-B are known to bind to two different tyrosine kinases receptors (VEGFR1 and VEGFR2) that are both expressed in nociceptors (Hulse et al., 2014; Selvaraj et al., 2015; Hamilton et al., 2016), and two neuropilin co-receptors (NRP-1 and NRP-2) that enhance the affinity of the ligands for the receptors. Additionally VEGF-A exists in alternative splice forms, VEGF-A_xxx_a or VEGF-A_xxx_b, xxx signifying the number of aminoacids implicated (Hulse et al., 2015; Peach et al., 2018). This alternative splicing is dependent on serine-arginine rich protein kinase 1 (SRPK1) which mediates the phosphorylation of serine-arginine rich splice factor (SRSF1). This phosphorylation leads to an increased production of VEGF-A_xxx_a (Hulse, 2017). Under certain pathological conditions including chronic pain, VEGF-A alternative splice forms appear to be dysregulated (Hulse, 2017).

VEGF-A_165_b is a VEGFR2 partial agonist that competes with VEGF-A_165_a for binding to VEGFR2. The VEGF-A_165_a isoform also binds to the NRP-1 co-receptor, whereas VEGF-A_165_b does not (Peach et al., 2018). The activation of distinct receptors implies different downstream cellular signal pathways and therefore different effects: VEGF-A_xxx_a seems to sensitize C nociceptors via Transient Receptor Potential (TRP) channels, namely TRPV1 and TRPA1, whereas VEGF-A_xxx_b exerts anti-nociceptive functions by suppressing the activity of those chanels (Hulse et al., 2014, 2015). Interestingly, although the angiogenic effect of VEGF-A is mediated by VEGFR2 (Ferrara et al., 2003), the direct nociceptive effect of VEGF-A appears to be mediated by VEGFR1 through TRPV1 trafficking and increased cell surface expression (Selvaraj et al., 2015). Furthermore, other ligands from VEGF family, VEGF-B and placental growth factor-2 are also able to directly stimulate nociceptors (Selvaraj et al., 2015).

VEGF receptors are abundantly expressed in humans and VEGF biology has been reported to be a complex issue in which the role of the co-receptors is poorly understood. Many studies which describe functional outcomes of VEGFR1 or VEGFR2 signaling do not clearly delineate the receptors and specific isoforms involved (Stuttfeld and Ballmer-Hofer, 2009; Rosenstein et al., 2010; Shibuya, 2011; Peach et al., 2018). Nociception seems to be one of these contexts and therefore, the current understanding and unknowns regarding VEGF signaling in nociception will be discussed below.

## Involvement of VEGF in several types of pain

Inflammation is a common feature in different painful syndromes and its components (inflammatory soup) sensitize nociceptors which mediate pain sensation. VEGF is one of the most important mediators participating in this pro-inflammatory milieu. The significance of VEGFR1 and VEGFR2 in the pathophysiology of two of the most prevalent chronic inflammatory diseases that are concurrent with pain, namely rheumatoid arthritis and osteoarthritis, has been previously reported (Hamilton et al., 2016). However, the role of each VEGF family member, isoforms or alternative splicing in alleviating chronic inflammatory pain have been poorly described.

In osteoarthritis (OA), anomalous VEGF expression in synovial fluids has been associated with higher pain scores (Takano et al., 2018) and worse prognosis. VEGF seems to mediate cartilage degeneration, bone and neurovascular invasion of articular cartilage, increased migration and/or activity of macrophages, fibroblasts, and neutrophils. These cells, in turn, increase levels of cytokines and VEGF, amplifying the inflammatory response (Hamilton et al., 2016; Nagao et al., 2017). VEGF is able to evoke pain by several pathways in synovium, osteochondral junction and meniscus, through both VEGFR1 and VEGFR2 (Nagao et al., 2017). Both signaling axes seem to be directly associated with nociceptor sensitization, and accordingly, VEGF signaling inhibition led to a decreased pain (Hamilton et al., 2016). In addition, other VEGF approaches have been experimentally tested and successfully counteracted pain responses and/or improved cartilage degeneration, synovitis and osteophyte formation (Nagai et al., 2014; Hamilton et al., 2016). Taking all of the aforementioned, it seems plausible that proper VEGF therapies targeting ligands or receptors could counteract osteoarthritis progression and its associated pain.

In other painful chronic diseases with an autoimmune component, such as rheumatoid arthritis (RA), one of the most potent factors that seems to be responsible for the typical hypertrophied synovium (pannus), oedema, swelling, and chondrolytic and osteolytic reactions, is VEGF (Afuwape et al., 2002; Malemud, 2007). This is expressed in synovial fibroblasts, fibroblasts close to microvessels, vascular smooth muscle and macrophages, but not in endothelial cells (Nagashima et al., 1995). VEGF is augmented in patients serum and is tightly correlated with TNF-α and some other pro-algesic cytokines (IL-1ß, IL-17, IL-18) which in turn reduce VEGF expression, except in patients who are refractory to TNF-α therapy (Nowak et al., 2008; Beazley-Long et al., 2018). At experimental level, an increased expression of VEGF, VEGFR1 and VEGFR2 was described in an RA animal model and the treatment with an anti-VEGFR1 efficiently blocked pain. However, the neutralization of either the VEGF ligand or VEGFR2 did not induce the same anti-nociceptive effect (De Bandt et al., 2003). Contrastingly, other authors suggested that VEGFR2 acts as a positive transducer in vascular proliferation during RA and its pharmacological blockade reduces mechanical sensitivity in an animal model of RA (Beazley-Long et al., 2018). While Beazley-Long and colleagues stated that when VEGFR2 is inhibited allodynia is reduced and/or prevented (Beazley-Long et al., 2018), De Bandt and colleagues showed VEGFR2 suppression was insufficient for resolution of this type of pain (De Bandt et al., 2003). Further studies aimed to address this discrepancy are needed.

The putative role of VEGF in the relief of pain has been most extensively studied in neuropathic pain compared to other types of pain. VEGF-A has been strongly linked with neuroprotection and its neutralization was found to exacerbate neuropathic damage and pain in a retrospective clinical study (Matsuoka et al., 2016). Contrary to this, experimental approaches of VEGF blockade have successfully alleviated nociceptive responses in a model of chronic constriction injury, sciatic nerve ligation or diabetic neuropathy. These approaches included the suppression of VEGFR2 signaling, spinal SRPK1 inhibition, and the administration of VEGF-A_xxx_b. The anti-nociceptive effect derived from VEGFR2 blockade in paiunful neuropathies has been reported to be mediated via the interaction with P2X_2/3_ receptors (De Bandt et al., 2003; Liu et al., 2012), or TRPA1 and/or TRPV1 (Hulse et al., 2015, 2016; Zeng et al., 2018). *In vitro* studies revealed that in injured peripheral nerves there is an upregulation of VEGF-A in infiltrated cells that seems to mediate angiogenesis, a key component of chronic inflammation and peripheral sensitization (Kiguchi et al., 2014). Blocking VEGF-A has been shown to reduce nociception in rodents and to exert a neuroprotective effect by improving neuronal restoration and conduction, decreasing pro-apoptotic Caspase-3 levels in sensory neurons, preventing neural perfusion and epidermal sensory fiber loss (Taiana et al., 2014; Hulse et al., 2015; Zeng et al., 2018). Another plausible strategy evaluated is SRPK1 inhibition, as this would reduce the pro-nociceptive and pro-angiogenic forms of VEGF (Hulse, 2017). Several studies indicate that administration of VEGF-A_165_b (the reported anti-nociceptive form of VEGF-A), could constitute an interesting therapeutic strategy for pain, considering that it also has neuroprotective effects. Contrastingly, a recent study demonstrated in an animal model of oxaliplatin-induced pain, that VEGF-A_165_b expression is augmented in spinal cord, and the intrathecal administration of bevacizumab or VEGF-A_165_b antibody reversed the hypersensitivity symptoms (Di Cesare Mannelli et al., 2018).

Most studies at an experimental level seem to suggest a pro-nociceptive effect induced by VEGF-A in several types of pain. However, in neuropathic pain Hulse and colleagues focused on the anti-nociceptive effect of VEGF-A_xxx_b isoform and the relevance of targeting its alternative splicing so as to modulate the balance between the pro- and anti-nociceptive VEGF isoforms. This had been shown extensively in several papers from their group (Hulse et al., 2014, 2015, 2016; Selvaraj et al., 2015; Hulse, 2017; Beazley-Long et al., 2018). However, in a model of oxaliplatin-induced pain, Di Cesare Mannelli and colleagues (Di Cesare Mannelli et al., 2018), clearly showed the pro-nociceptive role of VEGF-A_xxx_b isoform. Further studies are urgently needed in order to clarify the role of VEGF-A_xxx_b and the mechanisms underlying the paradoxical effects reported. These disparate functions raise the possibility that different isoforms may have varying pro- and anti-nociceptive role.

Among chronic neurologic diseases, migraine is the third most prevalent and disabling. Current treatments are usually unsuccessful. The meningeal and brain mast cells involved can degranulate and release vasoactive substances that can activate trigeminovascular mechanisms inducing pain. Among these mediators, VEGF is one of the most important as it also stimulates nitric oxide synthase and therefore increases nitric oxide levels (Bussolati et al., 2001). Therefore, VEGF plays a direct role in the endothelial cells in the trigeminovascular system. Indeed, increased levels of VEGF have been showed in migraneurs suggesting endothelial alterations (Rodríguez-Osorio et al., 2012). However, decreased serum concentrations of VEGF were found during interictal period (Michalak et al., 2017). In addition, several VEGF haplotypes have been described to be associated with variable susceptibility to migraine (Gonçalves et al., 2010). A better understanding of VEGF fluctuations, genetic profiling and the potential protective role in migraines could constitute an interesting approach for prophylactic intervention.

The importance of VEGF in cancer pathophysiology and therapy has been extensively reported (Carmeliet, 2005; Lal Goel and Mercurio, 2014). However, the potential anti-nociceptive effect of VEGF in cancer-induced pain is poorly understood. VEGFR1 is augmented in humans and in an animal model of osteosarcoma-induced pain. The modulation of VEGF-VEGFR1 axis signaling by an anti-VEGFR1 antibody or the administration of the VEGFR1 soluble form (sFLT1) that decoys VEGF from binding VEGFR1, effectively counteracted pain (Selvaraj et al., 2015). Lastly, only one study addressed the involvement of VEGF in neoplastic pain, the anti-nociceptive role derived from the inhibition of VEGF/VEGFR1, but not VEGFR2 (Selvaraj et al., 2015). Additional studies using experimental models of cancer-induced pain that address the role of VEGFR2 are urgently required in order to delineate the role for this integral mediator. This will inform the design and development of new pharmacological strategies.

In summary, although the role of VEGF and its receptors in some pathologies has been extensively studied, their function in nociception has not yet been fully elucidated. Current studies exemplify the successful alleviation of pain through the targeting of VEGF or VEGF receptors, but some considerations have to be taken:

- Although the majority of evidence supports the pro-nociceptive effects of VEGF-A, in neuropathic pain some authors have observed an anti-nociceptive effect of one VEGF-A splice form. It is therefore essential to understand alternative splice mechanisms, SRPK1 factor and the balance of each splice forms (VEGF-A_xxx_a/VEGF-A_xxx_b) to facilitate specific targeting and hence provide effective analgesia minimizing undesirable effects in sensory neurons.
- Considering the link between the aetiopathogenesis of some types of pain (i.e., endothelial alterations in migraine) and VEGF, the involvement of this factor in pain signaling should be urgently addressed.
- The role of other VEGF family members (i.e., VEGF-B) in nociception should be explored to clarify specific receptor-binding interactions and their corresponding molecular pathways. Together, these will allow us to unravel the mechanisms of previously reported unique functional outcomes.
- Due to their pivotal role in neuroprotection, the potential involvement of VEGF family members and alternative splicing forms should be investigated thorougly in neuropathic pain or multifactorial pain (e.g., cancer-related pain).

## Author contributions

ML-S and SG-R conceived the idea and wrote the manuscript.

### Conflict of interest statement

The authors declare that the research was conducted in the absence of any commercial or financial relationships that could be construed as a potential conflict of interest.

## Footnotes

^1^www.iasp-pain.org.
